# N^6^-methyladenosine-mediated LDHA induction potentiates chemoresistance of colorectal cancer cells through metabolic reprogramming

**DOI:** 10.7150/thno.73746

**Published:** 2022-06-13

**Authors:** Kun Zhang, Tao Zhang, Yuhan Yang, Wenling Tu, Hongbin Huang, Yujun Wang, Yuzhuo Chen, Kejian Pan, Zhuojia Chen

**Affiliations:** 1 The Second Affiliated Hospital of Chengdu Medical College, China National Nuclear Corporation 416 Hospital, Chengdu 610051, China.; 2 School of Biological Sciences and Technology, Chengdu Medical College, Chengdu 610500, China.; 3 Sun Yat-sen University Cancer Center; State Key Laboratory of Oncology in South China; Collaborative Innovation Center for Cancer Medicine, Guangzhou 510060, China.; 4 School of Pharmacy, Chengdu Medical College, Chengdu 610500, China.

**Keywords:** Glycolysis, METTL3, 5-FU, LDHA, Chemoresistance

## Abstract

**Background:** Chemoresistance to 5-fluorouracil (5-FU) is a major barrier to influence the treatment efficiency of colorectal cancer (CRC) patients, while the precise molecular mechanisms underlying 5-FU resistance remain to be fully elucidated.

**Methods:** The metabolic profiles including ATP generation, glucose consumption, lactate generation, and oxygen consumption rate (OCR) in 5-FU resistant CRC cells were compared with those in their parental cells. Subsequently, a series of *in vitro* and *in vivo* experiments were carried out to investigate the mechanisms responsible for metabolic reprogramming of 5-FU resistant CRC cells.

**Results:** We found that 5-FU resistant CRC cells showed increased levels of ATP generation, glucose consumption, lactate generation, and OCR as compared with those in their parental cells. Further, increased levels of mRNA N^6^-methyladenosine (m^6^A) and methyltransferase-like 3 (METTL3) were observed in 5-FU resistant CRC cells. Inhibition or knockdown of METTL3 can suppress glycolysis and restore chemosensitivity of 5-FU resistant CRC cells. Mechanistically, METTL3 enhances the expression of LDHA, which catalyzes the conversion of pyruvate to lactate, to trigger glycolysis and 5-FU resistance. METTL3 can increase the transcription of LDHA via stabilizing mRNA of hypoxia-inducible factor (HIF-1α), further, METTL3 also triggers the translation of LDHA mRNA via methylation of its CDS region and recruitment of YTH domain-containing family protein 1 (YTHDF1). Targeted inhibition of METTL3/LDHA axis can significantly increase the *in vitro* and *in vivo* 5-FU sensitivity of CRC cells.

**Conclusion:** Our study indicates that METTL3/LDHA axis-induced glucose metabolism is a potential therapy target to overcome 5-FU resistance in CRC cells.

## Introduction

Colorectal cancer (CRC), also known as large bowel cancer, is the second most common cause of cancer-related mortality worldwide 1. Despite the fact that targeted therapy and immunotherapy can benefit therapy outcomes, chemotherapy have been widely used and can significantly improve the prognosis of advanced and metastatic CRC patients 2. Further, 5-fluorouracil (5-FU), an apyrimidine analog that inhibits thymidylate synthase 3, is the first-line drug in the treatment of advanced and metastasis CRC^2^. Most patients will be exposed to multiple fluoropyrimidines (FPs)-based regimens administered sequentially. However, chemoresistance to 5-FU and other FPs is a major barrier to influence the treatment efficiency with adequate patients' outcomes 4. Although several studies have revealed that alterations in oncogenes and tumour suppressor genes contribute to chemoresistance and development of CRC 5, the precise molecular mechanisms underlying 5-FU resistant CRC remain to be fully elucidated.

Recent studies indicated that metabolic reprogramming plays a critical role in cancer malignancy including chemoresistance 6. Increased drug resistance is associated with reduced glucose levels and enhanced glycolysis phenotype 7. Upregulation of glycolytic enzymes were observed in human colon cancer cells with 5-FU resistance 8. Inhibition of glycolysis by targeting hexokinase II can sensitize human hepatocellular carcinoma cells to 5-FU treatment 9. Alteration of glucose metabolism was able to re-sensitize gastric cancer cells with hypoxia-induced resistance to 5-FU 10.

As to the glycolysis pathway, glucose transporter (GLUT) proteins were responsible for glucose entry into the cell. Then, glucose is phosphorylated by hexokinases (HKs) and remains trapped inside the cell. Through glycolysis, glucose is metabolized to the final product pyruvate. As a result, lactate dehydrogenase (LDH) catalyzes pyruvate to lactate instead of acetyl-CoA, which can otherwise be used as TCA cycle intermediate 11. Both LDHA and LDHB can catalyze pyruvate to lactate reaction 12. The expression of glycolytic enzymes (e.g., GLUTs and LDHA) was upregulated in chemoresistant cells 13. Suppression of glucose metabolism by targeting LDHA can sensitize cancer cells to cisplatin treatment 14. Consistently, long noncoding RNA HAGLR promotes 5-Fu resistance in gastric cancer through targeting the LDHA-glycolysis pathway 15. All these results suggested that glycolysis and glycolytic enzymes such as LDHA might be key factors regulating chemoresistance of cancer cells.

Epigenetic reprogramming plays a key role in the acquisition of chemoresistant potential of cancer cells 16. *N*^6^-methyladenosine (m^6^A) is the most abundant modification in human mRNA which can regulate mRNA splicing, decay and translation 17. Recent data indicated that m^6^A can accelerate the Warburg effect to induce cancer cell growth and metastasis 18, 19. For example, R-2-hydroxyglutarate attenuates aerobic glycolysis in leukemia by targeting the FTO/m^6^A/PFKP/LDHB axis 20. LncRNA LINRIS can stabilize IGF2BP2 to promote the aerobic glycolysis in CRC cells 21. Further, methyltransferase-like 3 (METTL3) can stabilize HK2 and SLC2A1 (GLUT1) expression in CRC through an m^6^A-IGF2BP2/3-dependent mechanism to trigger glycolysis and cell growth 19. Recent study indicated that pyruvate dehydrogenase kinase 4 (PDK4) is involved in m^6^A-regulated glycolysis and ATP generation 22. In addition, m^6^A demethylase ALKBH5 can modulate CK2-mediated glycolysis to regulate cisplatin sensitivity of cancer cells 23. All these results suggested that m^6^A and its related regulators might regulate glycolysis and chemosensitivity in CRC cells.

Our present study revealed significant alteration in metabolic profiling including ATP generation, glucose consumption, lactate production in 5-FU resistant CRC cells. Further, there were increased levels of m^6^A in 5-FU resistant cells, while METTL3-deleted cells were more sensitive to 5-FU treatment. Mechanistical investigations showed that METTL3 increased the translation efficiency and transcription of LDHA to trigger glycolysis and chemoresistance of CRC cells.

## Results

### The metabolic reprogramming of 5-FU resistant CRC cells

**Figure 1:**
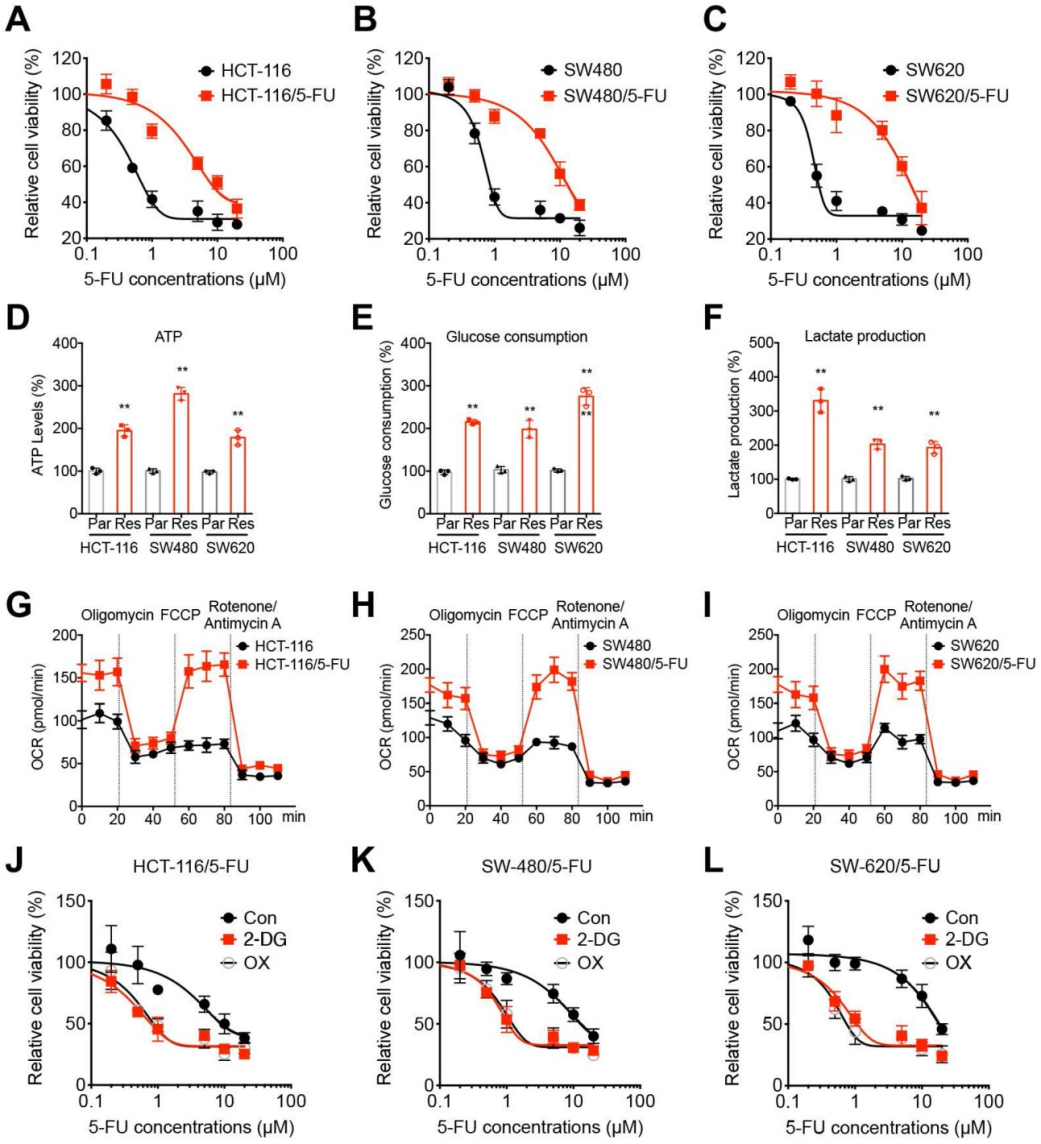
** 5-FU resistant OS cells showed metabolic reprogramming. (A-C)** The 5-FU sensitivity of HCT-116/5-FU(A), SW480/5-FU (B), and SW620/5-FU (C) cells and parental cells after treated with increasing concentrations of 5-FU for 24 h. **(D-F)** The ATP generation (D), glucose consumption (E), and generation of lactate (F) in CRC parental and 5-FU resistant cells. **(G-I)** Variation of OCR was determined in HCT-116/5-FU(G), SW480/5-FU (H), SW620/5-FU (I) cells and parental cells, respectively. Experiments were performed in six replicates. **(J-L)** HCT-116/5-FU(J), SW480/5-FU (K), SW620/5-FU (L) cells were pretreated with 2-DG (10 mM) or OX (10 mM) for 90 min and then treated with increasing concentrations of 5-FU for 24 h. Data are presented as means ± SD of three independent experiments. ** p < 0.01 compared with control; NS, no significant.

Firstly, the 5-FU sensitivity of CRC parental and 5-FU resistant cells were evaluated by use of CCK-8 kits. Our data showed that the established 5-FU resistant cells were much more resistant to 5-FU treatment as compared to their corresponding parental cells. The IC_50_ values of 5-FU for HCT-116/5-FU and HCT-116 were 9.59 and 1.55 μM (Figure 1A), for SW480/5-FU and SW480 were 13.5 and 1.81 μM (Figure 1B), and for SW620/5-FU and SW620 were 14.8 and 1.21 μM (Figure 1C), respectively.

Chemoresistant cancer cells exhibited different properties such as mitochondrial respiration, oxidative phosphorylation, and aerobic glycolysis 24, 25. We then compared the metabolic profiling of chemoresistant and parental CRC cells. The results indicated that in CRC/5-FU cells, the ATP generation (Figure 1D), glucose consumption (Figure 1E), and lactate production (Figure 1F) were significantly increased as compared with those in parent CRC cells. Seahorse analysis indicated that the basal and maximal oxygen consumption rate (OCR) were elevated in 5-FU resistant CRC cells (Figure 1G-I) as compared with those in their parental cells, respectively. It indicated that 5-FU resistant CRC cells showed increased levels of glycolysis and OCR.

In order to evaluate the potential roles of glycolysis in 5-FU resistant CRC cells, cells were pretreated with glycolysis inhibitors 2-deoxy-D-glucose (2-DG) or oxamate (OX) and then incubated with 5-FU. Our results showed that glycolysis inhibitors 2-DG and OX can significantly restore the 5-FU sensitivity of all CRC/5-FU cells (Figure 1J, K, and L). All these data indicated that 5-FU resistant CRC cells showed increased levels of glycolysis, further, inhibition of glycolysis can restore the 5-FU sensitivity.

## m^6^A regulated metabolic reprogramming of 5-FU resistant CRC cells

**Figure 2:**
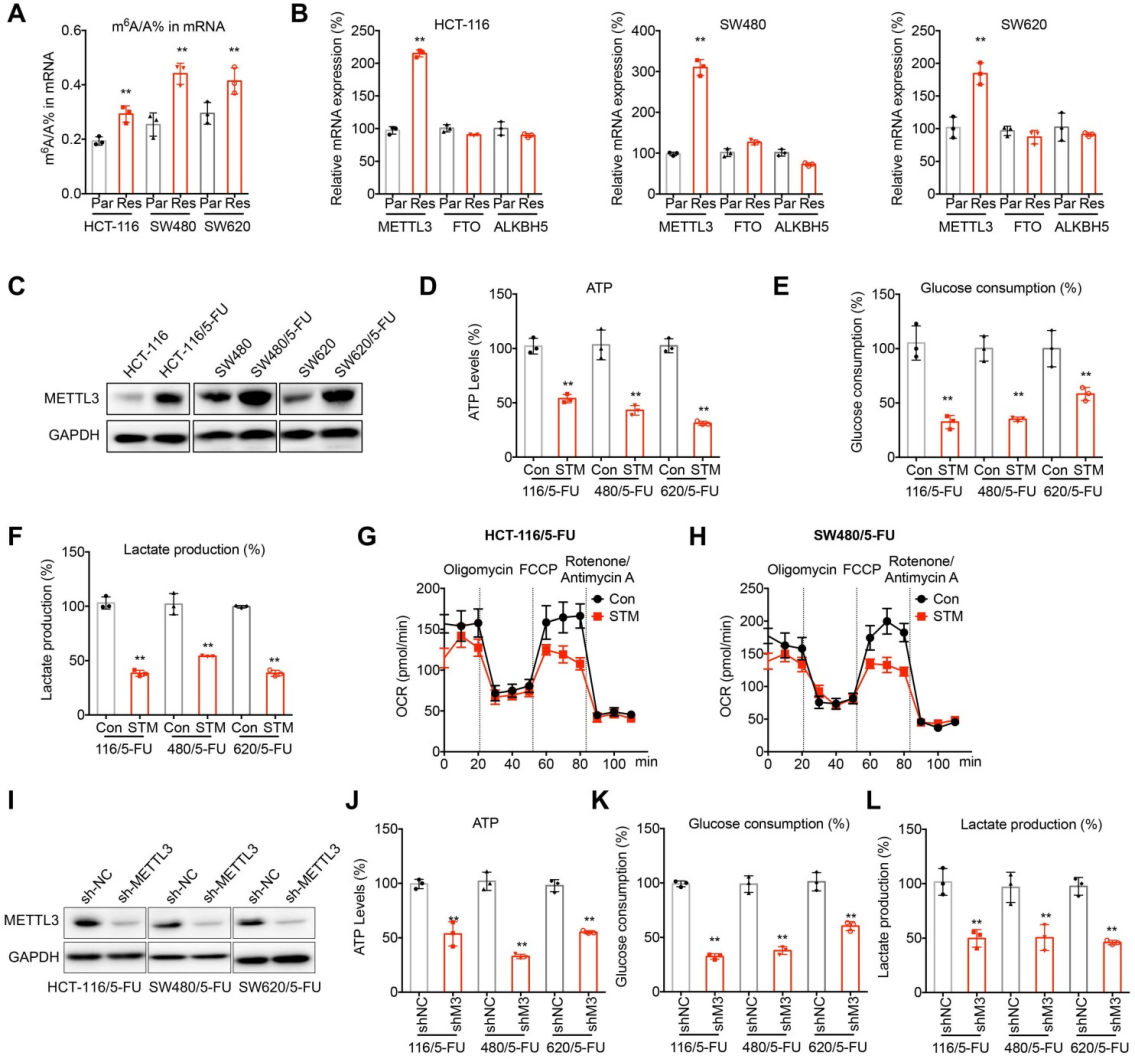
** m^6^A regulated metabolic reprogramming of 5-FU resistant CRC cells. (A)** The m^6^A/A ratio of total mRNA were determined by LC-MS/MS in CRC parental and 5-FU resistant cells. **(B)** The mRNA expression of m^6^A methyltransferases and demethylases in CRC parental and 5-FU resistant cells. **(C)** The expression of METTL3 in CRC parental and 5-FU resistant cells was checked by western blot analysis. **(D-F)** The ATP generation (D), consumption of glucose (E), and production of lactate production (F) in 5-FU resistant CRC cells treated with STM2457 (1 µM) for 24 h. **(G-H)** The variation of OCR was determined in HCT-116/5-FU (G) and SW480/5-FU (H) cells treated with STM2457 (1 µM) for 24 h. Experiments were performed in six replicates. **(I)** Levels of METTL3 in 5-FU resistant CRC cells transfected with sh-NC or sh-METTL3. **(J-L)** The ATP generation (J), consumption of glucose (K), and production of lactate production (L) in sh-NC or sh-METTL3 CRC/5-FU cells transfected with. Data are presented as means ± SD of three independent experiments. ** p < 0.01 compared with control; NS, no significant.

Considering that 5-FU resistant CRC cells acquired chemoresistant properties from parental cells, epigenetic reprogramming such as RNA modification may be involved in this process. Firstly, we checked the m^6^A levels of mRNAs in 5-FU resistant cells and their corresponding parental cells. The results showed that the m^6^A levels of mRNAs isolated from 5-FU resistant CRC cells were statistically (p < 0.05, *t*-test) greater than those of their corresponding parental cells (Figure 2A). Then the expression levels of m^6^A methyltransferases METTL3 and demethylases FTO and ALKBH5 were checked. qRT-PCR analysis showed that the mRNA of METTL3 was increased in all measured 5-FU resistant CRC cells (Figure 2B). Consistently, western blot analysis confirmed that the expression of METTL3 was upregulated in 5-FU resistant CRC cells (Figure 2C).

We therefore investigated whether METTL3 regulates metabolic reprogramming of 5-FU resistant CRC cells. Treatment with STM2457, an inhibitor of METTL3, significantly decreased the ATP generation (Figure 2D), glucose consumption (Figure 2E), production of lactate (Figure 2F) in 5-FU resistant CRC cells. Further, STM2457 treatment suppressed the basal and maximal OCR in 5-FU resistant CRC cells (Figure 2G and H).To confirm the essential roles of METTL3, we knocked down METTL3 in 5-FU resistant CRC cells (Figure 2H). Consistently, knockdown of METTL3 can inhibit the ATP generation (Figure 2I), glucose consumption (Figure 2J), and production of lactate (Figure 2K) in 5-FU resistant CRC cells. These results confirmed that m^6^A and METTL3 were involved in metabolic reprogramming of 5-FU resistant CRC cells.

## LDHA mediates METTL3-regulated metabolic reprogramming in 5-FU resistant cells

**Figure 3:**
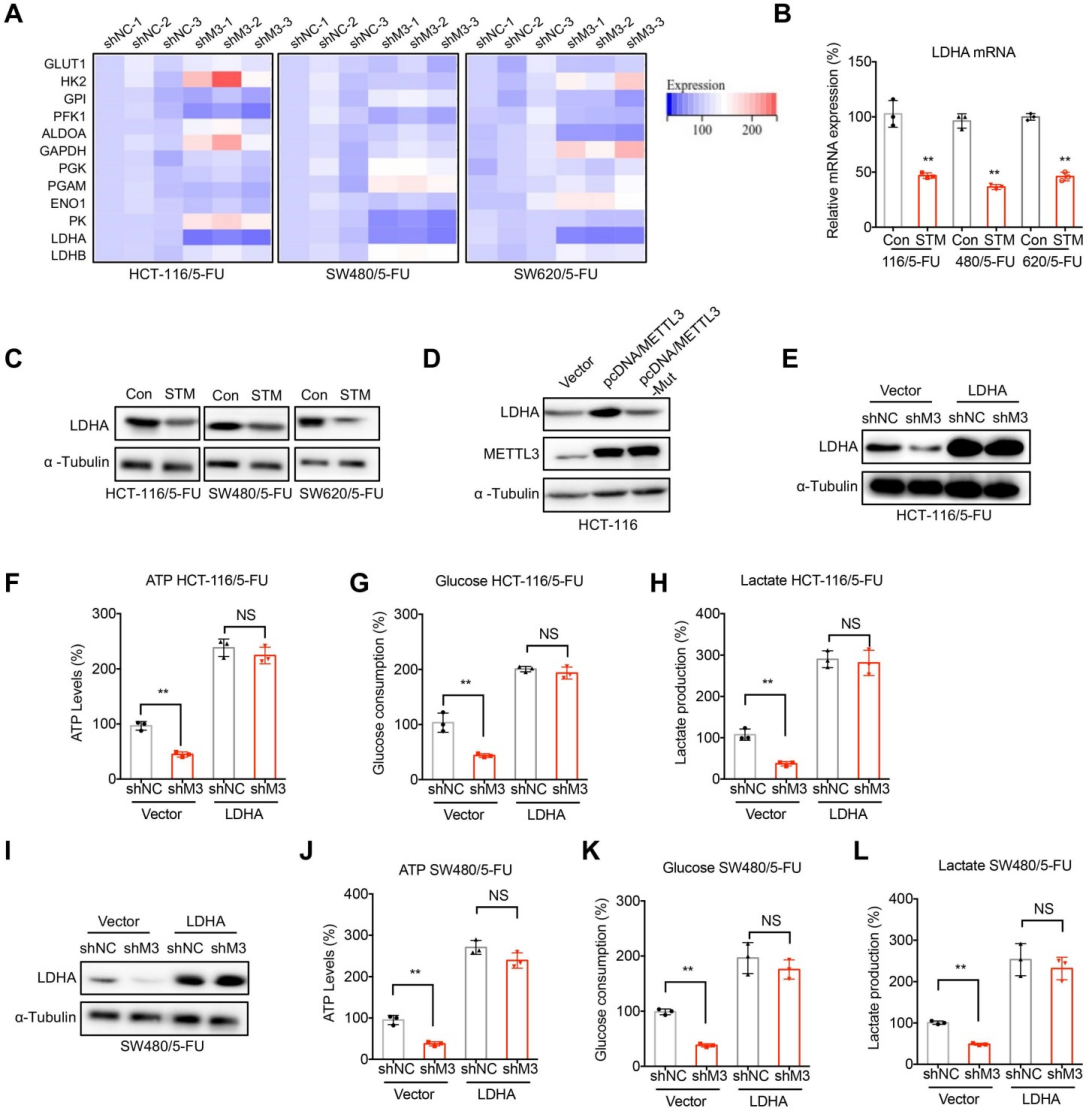
** LDHA mediates METTL3-regulated metabolic reprogramming in 5-FU resistant cells. (A)** The mRNA expression profiles of critical enzymes for glycolysis in sh-NC or sh-METTL3 CRC/5-FU cells. **(B & C)** The mRNA (B) and protein (C) expression of LDHA in 5-FU resistant CRC cells treated with STM2457 (1 µM) for 24 h. **(D)** HCT-116 cells were transfected with pcDNA vector, pcDNA/METTL3, or pcDNA/METTL3-Mut for 24 h, the levels of LDHA and METTL3 were checked. **(E-H)** sh-NC or sh-METTL3 HCT-116/5-FU cells were transfected with pcDNA vector or pcDNA/LDHA for 24 h, the expression of LDHA was checked (E), and then the ATP generation (F), consumption of glucose (G), and production of lactate production (H) were measured. **(I-L)** sh-NC or sh-METTL3 SW480/5-FU cells were transfected with pcDNA vector or pcDNA/LDHA for 24 h, the expression of LDHA was checked (I), and then the ATP generation (J), consumption of glucose (K), and production of lactate production (L) were measured. Data are presented as means ± SD of three independent experiments. ** p < 0.01 compared with control; NS, no significant.

GLUT1/2, HK2, GPI, PFK1, ALDOA, GAPDH, PGK, PGAM, ENO1, PK, and LDHA/B are critical enzymes for glycolysis in cancer cells 26. We then checked the mRNA expression levels of these key enzymes in sh-NC or sh-METTL3 CRC/5-FU cells. Our results showed that LDHA, which catalyzes the conversion of pyruvate into lactate, was significantly decreased in sh-METTL3 CRC/5-FU cells (Figure 3A). Further, STM2457 treatment suppressed the mRNA (Figure 3B) and protein (Figure 3C) expression of LDHA in all measured CRC/5-FU cells. In addition, over expression of METTL3 can increase the protein expression of LDHA in HCT-116 cells, however, enzyme inactivated METTL3 had no similar effect (Figure 3D). These results suggested that METTL3 regulated the expression of LDHA via an m^6^A enzyme activity dependent manner.

We therefore investigated whether LDHA was involved in METTL3-regulated metabolic reprogramming in 5-FU resistant cells. Our results showed that over-expression of LDHA (Figure 3E) can significantly reverse sh-METTL3-suppressed ATP generation (Figure 3F), glucose consumption (Figure 3G), production of lactate (Figure 3H) in HCT-116/5-FU cells. Consistently, over expression of LDHA also reversed sh-METTL3-suppressed ATP generation (Figure 3I), glucose consumption (Figure 3J), production of lactate (Figure 3K) in 5-FU resistant SW480 cells. These results confirmed that LDHA was involved in METTL3-regulated metabolic reprogramming in 5-FU resistant cells.

## METTL3 regulated transcription and translation of LDHA in 5-FU resistant cells

**Figure 4:**
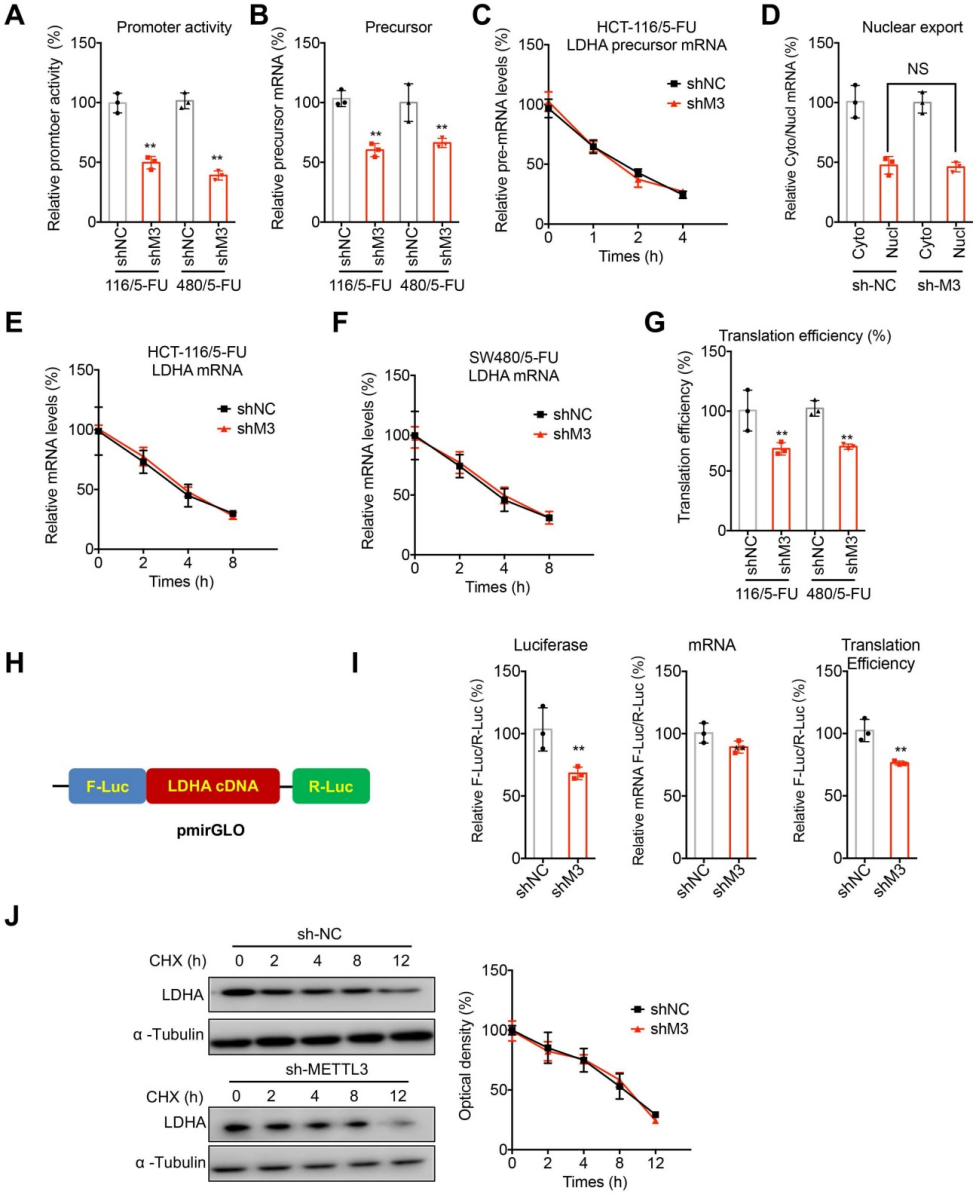
** METTL3 regulated the transcription and translation of LDHA in 5-FU resistant cells. (A)** Cells were transfected with pGL3-LDHA-luc reporter and pRL-TK plasmid for 24 h. Results were expressed as the ratios between the activity of the reporter plasmid and pRL-TK. **(B)** The levels of precursor mRNA in sh-NC or sh-METTL3 CRC/5-FU cells. **(C)** sh-NC or sh-METTL3 HCT-116/5-FU cells were pre-treated with Act-D for 90 min, then precursor mRNA of LDHA was analyzed at indicated times. **(D)** The relative levels of nuclear versus cytoplasmic LDHA mRNA in sh-NC or sh-METTL3 HCT-116/5-FU cells. **(E & F)** sh-NC and sh-METTL3 HCT-116/5-FU (E) or SW480/5-FU cells were pre-treated with Act-D for 90 min, then mature mRNA of LDHA was analyzed at indicated times. **(G)** The translation efficiency of endogenous LDHA in was checked in sh-NC and sh-METTL3 HCT-116/5-FU by normalization of ATP5D protein levels to the relative mRNA abundance. **(H)** Schematic representation of LDHA cDNA of pmirGLO vector to investigate the roles of METTL3 in LDHA translation. **(I)** sh-NC and sh-METTL3 HCT-116/5-FU cells were transfected with pmirGLO-LDHA reporter for 24 h. The translation outcome was determined as a relative signal of F-luc divided by R-luc, the mRNA abundance was determined by qRT-PCR of F-luc and R-luc, and the translation efficiency of LDHA is defined as the quotient of reporter protein production (F-luc/R-luc) divided by mRNA abundance 28. **(J)** sh-NC and sh-METTL3 HCT-116/5-FU cells were treated with CHX for the indicated times, and protein expression of LDHA was analyzed by western blot analysis (*left*) and quantitatively analyzed (*right*). Data are presented as means ± SD from three independent experiments. *p < 0.05, NS, no significant, by Student's* t* test.

We further investigated potential mechanisms responsible for METTL3-regulated expression of LDHA in 5-FU resistant CRC cells. Since mRNA levels of LDHA were significantly decreased in sh-METTL3 5-FU resistant CRC cells, we checked the effects of sh-METTL3 on transcription, precursor mRNA splicing, and nuclear to cytoplasm exporting in HCT-116/5-FU and SW480/5-FU cells. Firstly, the promoter region of LDHA was sub-cloned into pGL3 basic plasmid to generate promoter activity reporter. Dual luciferase assay showed that promoter activities of LDHA in both sh-METTL3 HCT-116/5-FU and SW480/5-FU cells were significantly less than that in sh-NC cells (Figure 4A), suggesting that METTL3 can regulate the transcription of LDHA. Consistently, levels of precursor mRNA of LDHA were decreased in sh-METTL3 HCT-116/5-FU and SW480/5-FU cells as compared with those in sh-NC cells (Figure 4B). Then, we checked the splicing rate of precursor mRNA via treating cells with Act-D to measure the abundance of pre-mRNA 27. Results showed that splicing rate of LDHA was comparable between sh-NC and sh-METTL3 HCT-116/5-FU cells (Figure 4C). Further, nuclear and cytoplasmic RNAs were extracted from their corresponding fractions. There was no significant variation for the relative abundance of LDHA in nucleus and cytoplasm of sh-NC and sh-METTL3 HCT-116/5-FU cells (Figure 4D). These data indicated that METTL3 can positively regulate the transcription of LDHA.

It has been revealed that m^6^A can directly regulate the mRNA stability and translation of mRNA 28, 29. Our data showed that mature mRNA stability of LDHA had no significant variation between sh-NC and sh-METTL3 HCT-116/5-FU cells (Figure 4E). Similar results were also observed in SW480/5-FU cells (Figure 4F). As to the translation efficiency, we measured its endogenous translation efficiency via dividing the quotient of protein production (LDHA/α-tubulin) by mRNA abundance 28. Our data showed that knockdown of METTL3 significantly decreased the translation efficiency of LDHA in both HCT-116/5-FU and SW480/5-FU cells (Figure 4G). To confirm the effect of METTL3 on translation of LDHA, LDHA cDNA including 5'UTR, CDS and 3'UTR was sub-cloned into pmirGLO to generate luciferase reporter (Figure 4H). Our data showed that sh-METTL3 can significantly decrease the translation efficiency of pmirGLO-LDHA in HCT-116/5-FU cells (Figure 4I).

To investigate that whether METTL3 can directly or indirectly regulate LDHA protein stability, we treated both sh-NC and sh-METTL3 cells with cycloheximide (CHX), which can block protein translation. Results showed that there is no significant variation for protein stability of LDHA in sh-NC and sh-METTL3 cells (Figure 4J).

Collectively, our data suggested that METTL3 regulated the transcription and translation of LDHA in 5-FU resistant cells.

## HIF-1α is involved in METTL3-regulated transcription of LDHA

**Figure 5:**
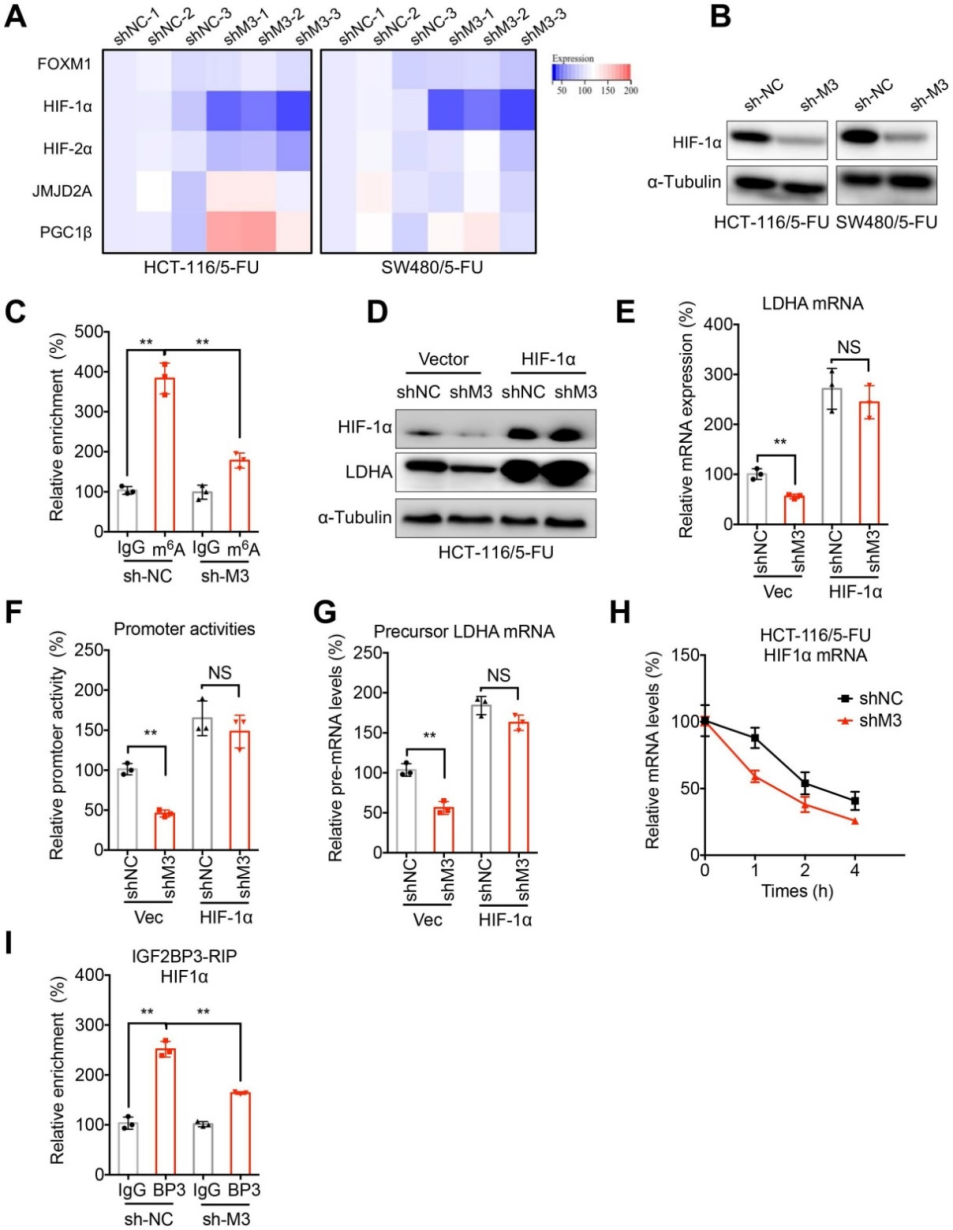
** HIF-1α is involved in METTL3-regulated transcription of LDHA. (A)** The mRNA expression profiles of transcription factors for LDHA in sh-NC or sh-METTL3 HCT-116/5-FU and SW480/5-FU cells. **(B)** The protein expression of HIF-1α in sh-NC or sh-METTL3 HCT-116/5-FU and SW480/5-FU cells. **(C)** m^6^A RIP-qPCR analysis of HIF-1α mRNA in sh-NC or sh-METTL3 CRC/5-FU cells. **(D & E)** sh-NC or sh-METTL3 HCT-116/5-FU were transfected with pcDNA and pcDNA/ HIF-1α for 24 h, the protein (D) and mRNA (E) of LDHAA was checked. **(F & G)** The promoter activities (F) and precursor mRNA (G) of LDHA in sh-NC or sh-METTL3 HCT-116/5-FU transfected with pcDNA and pcDNA/ HIF-1α for 24 h. **(H)** sh-NC and sh-METTL3 HCT-116/5-FU cells were pre-treated with Act-D for 90 min, then mature mRNA of HIF-1α was analyzed at indicated times. **(I)** RIP-qPCR analysis of HIF-1α mRNA in sh-NC and sh-METTL3 HCT-116/5-FU cells by use of antibody of IGF2BP3. Data are presented as the mean ± SD from three independent experiments. **p < 0.01. NS, no significant.

Mechanisms responsible for METTL3-regulated transcription of LDHA in 5-FU resistant CRC cells were further investigated. Since m^6^A may not directly regulate the transcription initiation, we checked the effects of sh-METTL3 on the transcription factors for LDHA. Transcriptionally, LDHA is regulated by forkhead box protein M1 (FOXM1), hypoxia-inducible factors (HIF-1α and HIF-2α), Jumonji C Domain 2A (JMJD2A), and peroxisome proliferator-activated receptor gamma (PPAR-γ) coactivator 1-beta (PGC1β) in cancer cells 12. Our data showed that HIF-1α was significantly decreased in sh-METTL3 HCT-116/5-FU and SW480/5-FU cells (Figure 5A). Further, protein expression of HIF-1α was also decreased in sh-METTL3 CRC/5-FU cells (Figure 5B). Further, m^6^A-RIP-PCR showed that mRNA of HIF-1α was significantly methylated by m^6^A in HCT-116/5-FU cells, while sh-METTL3 decreased the enrichment of m^6^A of HIF-1α mRNA (Figure 5C).

Although promotion of LDHA transcription by HIF1 has been revealed to be enhanced when cAMP binds to the cAMP response element (CRE) in the LDHA promoter region 30, we further verified its role in METTL3-regulated transcription of LDHA. Our data showed that over-expression of HIF-1α (Figure 5E) can reverse sh-METTL3-suppressed mRNA (Figure 5F) and protein (Figure 5E) expression of LDHA in HCT-116/5-FU cells. Further, luciferase assay confirmed that over-expression of HIF-1α can reverse the down regulation promoter activity of LDHA in sh-METTL3 HCT-116/5-FU cells (Figure 5F). Consistently, the decreased levels of precursor mRNA of LDHA in sh-METTL3 HCT-116/5-FU were also reversed after transfection of HIF-1α plasmid (Figure 5G). All these data confirmed that HIF-1α is involved in METTL3-regulated transcription of LDHA.

We further examined the mechanisms how METTL3 regulates HIF-1α. The results showed that knockdown of METTL3 can significantly decrease the half-life times of HIF-1α mRNA in HCT-116/5-FU cells (Figure 5H). It might be due to that sh-METTL3-decreased m^6^A of HIF-1α mRNA can impair the binding between IGF3BP3 and HIF-1α mRNA (Figure 5I), which can stabilize HIF-1α mRNA in human cancer cells 31.

## m^6^A methylation at CDS of LDHA mediates METTL3-regulated translation of LDHA

**Figure 6:**
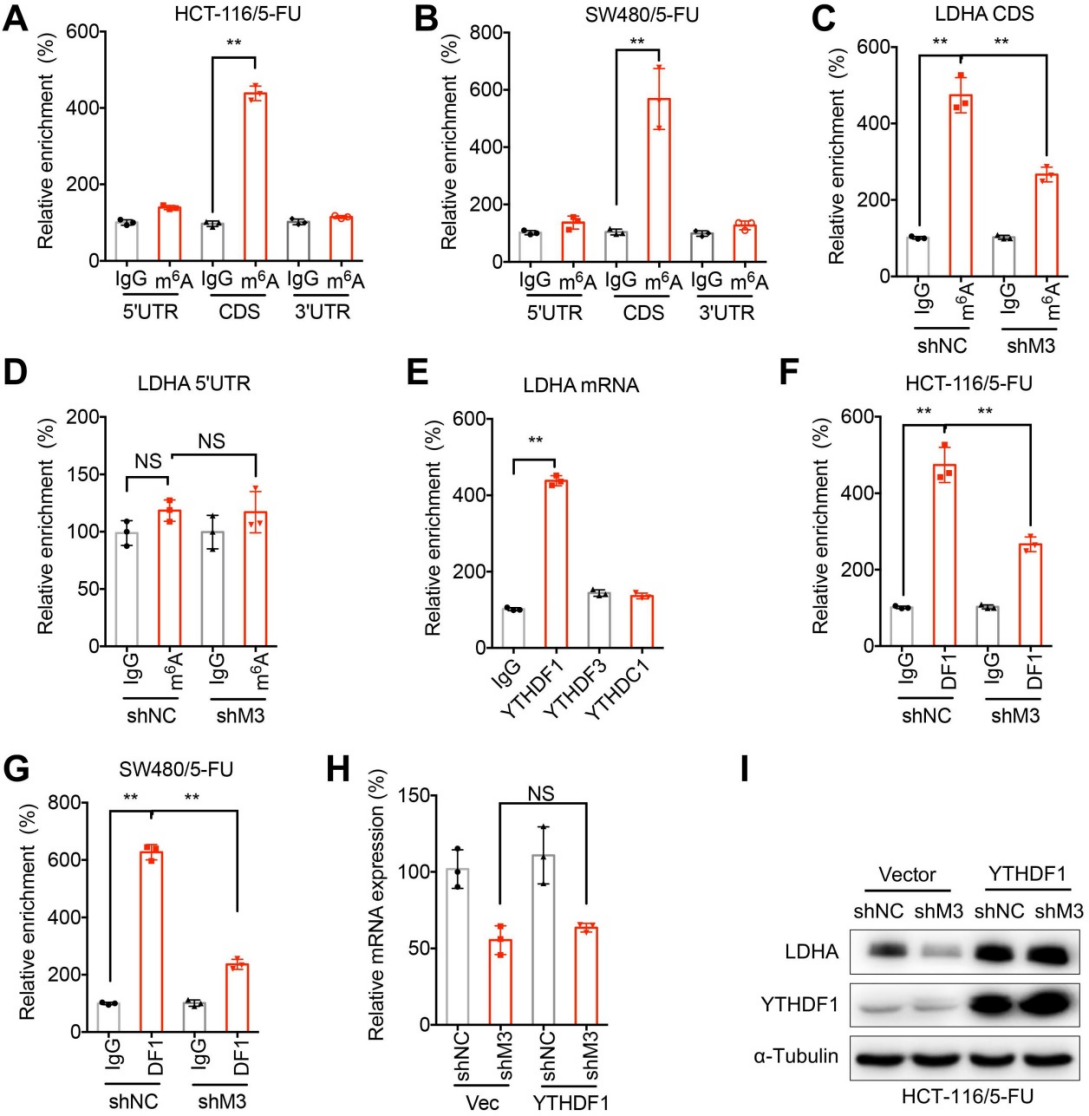
** m^6^A methylation at CDS of LDHA mediates METTL3-regulated translation of LDHA. (A & B)** The m^6^A in 5'UTR, CDS, or 3'UTR of LDHA in HCT-116/5-FU (A) or SW480/5-FU (B) cells were analyzed by m^6^A-RIP-qPCR using fragmented RNA. **(C & D)** The m^6^A in CDS (C) or 5'UTR (D) in LDHA mRNA from sh-NC or sh-METTL3 HCT-116/5-FU cells were checked by m^6^A-RIP-qPCR using fragmented RNA. **(E)** RIP-qPCR analysis of LDHA in HCT-116/5-FU cells by use of antibody of YTHDF1, YTHDF3, and YTHDC1. **(F & G)** RIP-qPCR analysis of LDHA in sh-NC or sh-METTL3 HCT-116/5-FU (F) or SW480/5-FU (G) cells were analyzed by use of antibody of YTHDF1. **(H & I)** The mRNA (H) and protein (I) of LDHA in sh-NC or sh-METTL3 HCT-116/5-FU transfected with pcDNA and pcDNA/YTHDF1 for 24 h. Data are presented as the mean ± SD from three independent experiments. **p < 0.01. NS, no significant.

We further investigated the methylation site of METTL3-regulated translation of LDHA. m^6^A-RIP-PCR with fragmented RNA indicated that only the CDS region of LDHA was significantly enriched by m^6^A antibody in both HCT-116/5-FU (Figure 6 A) and SW480/5-FU (Figure 6B) cells. Further, sh-METTL3 significantly decreased the m^6^A enrichment of *LDHA*-CDS (Figure 6C), while had no similar effect on 5'UTR (Figure 6D). It suggested that m^6^A methylation was at CDS of LDHA mRNA rather than at 5'UTR or 3'UTR.

We then investigated mechanisms responsible for m^6^A-regulated translation of LDHA. It has been reported that YTHDF1/3 and YTHDC1 can regulate translation of m^6^A methylated mRNA 28, 32. By use of RIP-PCR, our data showed that YTHDF1, while not YTHDF3 or YTHDC1, can significantly bind with *LDHA* mRNA in HCT-116/5-FU cells (Figure 6E). Further, knockdown of METTL3 can significantly decrease the binding between YTHDF1 and *LDHA* mRNA in both HCT-116/5-FU (Figure 6F) and SW480/5-FU (Figure 6G) cells. In order to confirm whether YTHDF1 mediates METTL3-regulated translation of LDHA, both sh-NC and sh-METTL3 HCT-116/5-FU cells were transfected with YTHDF1 plasmid. Over-expression of YTHDF1 had limited effect on mRNA of *LDHA* (Figure 6H), however, over expression of YTHDF1 can significantly attenuate sh-METTL3-suppessed protein expression of LDHA in HCT-116/5-FU cells (Figure 6I). All these data suggested that YTHDF1 is responsible for m^6^A-regulated translation of LDHA.

## METTL3/LDHA-regulated metabolic reprogramming promotes 5-FU resistance

**Figure 7:**
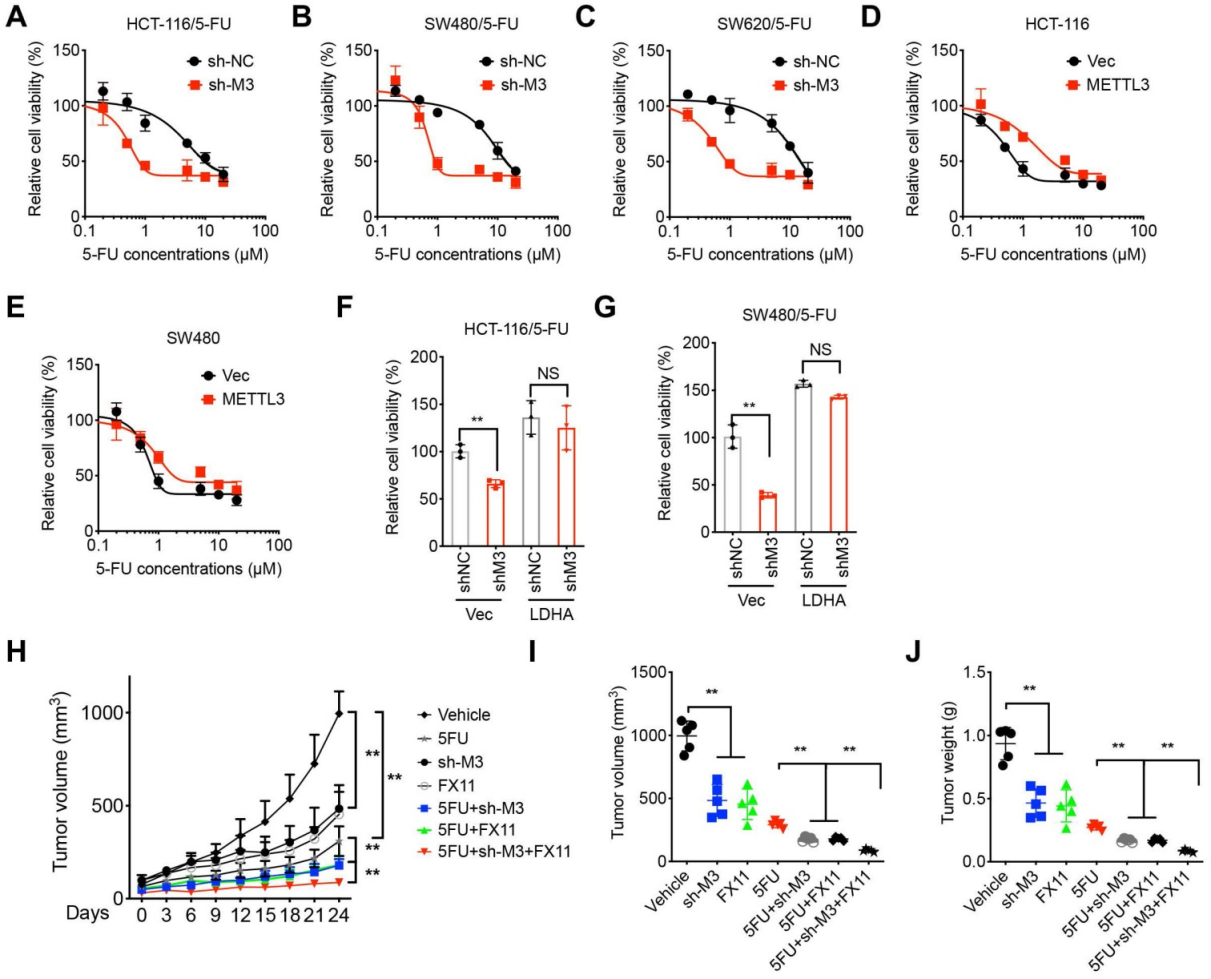
** METTL3/LDHA-regulated metabolic reprogramming promotes 5-FU resistance. (A-C)** Cell viability of sh-Control and sh-METTL3 HCT-116/5-FU (A), SW480/5-FU (B), and SW620/5-FU (C). **(D & E)** HCT-116 (E) or SW480 (F) cells were pre-transfected with pcDNA vector or pcDNA/METTL3 for 24 h and then further treated with 5-FU for 24 h. **(F & G)** sh-Control and sh-METTL3 HCT-116/5-FU (F) and SW480/5-FU (G) were pre-transfected with pcDNA vector or pcDNA/LDHA for 24 h and then further treated with 1 µM 5-FU for 24 h. **(H-J)** Xenografts of sh-Control and sh-METTL3 HCT-116/5-FU cells were treated with 5-FU combined with or without FX11. (H) The tumor growth curves were recorded every three days; Tumor volume (I) and weight (J) of xenografts for each group at the end of experiments. **p < 0.01. NS, no significant.

We investigated the roles of METTL3/LDHA axis- regulated metabolic reprogramming in 5-FU resistance of CRC cells. To verify the potential roles of m^6^A in the chemosensitivity of CRC cells, we knocked down METTL3 in 5-FU resistant CRC cells. Results showed that knockdown of METTL3 can restore the 5-FU sensitivity of HCT-116/5-FU (Figure 7A), SW480/5-FU (Figure 7B), and SW620/5-FU (Figure 7C) cells. In addition, over expression of METTL3 can decrease the *in vitro* 5-FU sensitivity of HCT-116 and SW480 cells, however, enzyme inactivated METTL3 had no similar effect (Figure 7D and E).

We further checked whether LDHA was involved in METTL3-regulated 5-FU sensitivity. Our data showed that over-expression of LDHA can reverse sh-METTL3-increased 5-FU sensitivity of both HCT-116/5-FU (Figure 7F) and SW480/5-FU (Figure 7G). It confirmed that LDHA was involved in METTL3 regulated 5-FU sensitivity. To verify the *in vivo* effects, mice were implanted with sh-control or sh-METTL3 HCT-116/5-FU cells and then further treated with or without 5-FU combined with or without LDH inhibitor FX11 33. Both sh-METTL3 and FX11 can increase* in vivo* 5-FU sensitivity, while sh-METTL3 and FX11 can synergistically increase *in vivo* 5-FU sensitivity of xenografts (Figure 7H). Further, tumor volume and weight in sh-METTL3 and FX11 group were significantly less than that of sh-METTL3 or FX11 alone group (Figure 7I and J). This result suggested that METTL3/LDHA axis regulated 5-FU resistance of CRC cells.

## Clinical characteristics of METTL3/LDHA axis on CRC progression

**Figure 8:**
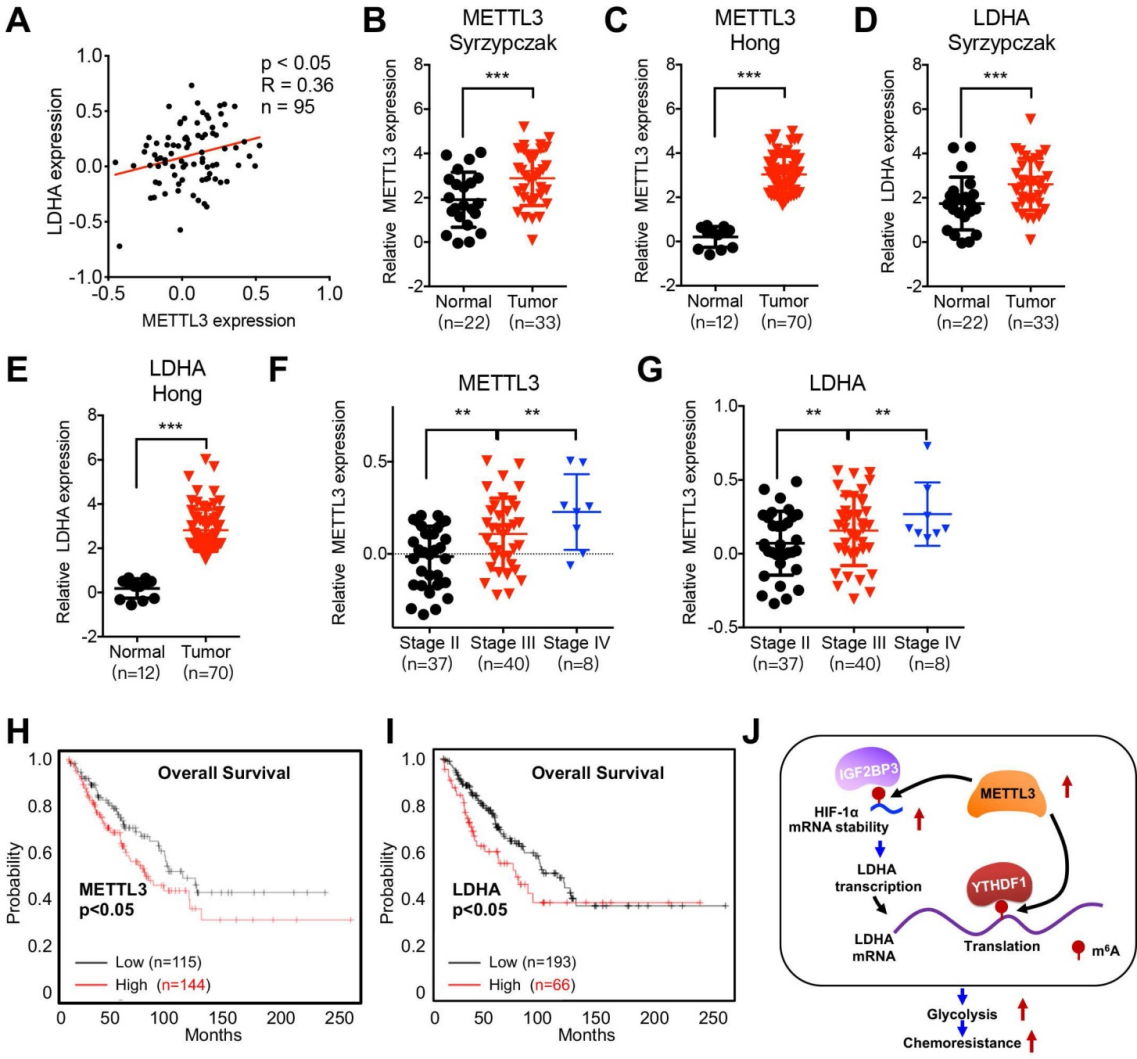
** Clinical characteristics of METTL3/LDHA axis on CRC progression. (A)** The correlation of METTL3 and LDHA in 95 CRC tissues was analyzed. **(B & C)** METTL3 mRNA expression in CRC tumor tissues and normal tissues from Oncomine database of Skrzypczak (B) and Hong (C) CRC. **(D & E)** LDHA mRNA expression in CRC tumor tissues and normal tissues from Oncomine database of Skrzypczak (D) and Hong (E) CRC. **(F & G)** Relative protein expression of METTL3 (F) and LDHA (G) in stage II/III/IV CRC tissues based on data available from the CPTAC database. **(H)** OS in CRC patient with high (n=144) vs. low (n=115) levels of METTL3 was plotted by the Kaplan-Meier method. **(I)** OS in CRC patient with high (n=193) vs. low (n=66) levels of LDHA was plotted by the Kaplan-Meier method. **(J)** Proposed model to illustrate the mechanisms of METTL3/LDHA axis to trigger the glycolysis and chemoresistance of CRC cells.

We further investigated the clinical characteristics of METTL3/LDHA axis on clinical CRC progression. The protein expression of METTL3 was positively correlated with the LDHA in CRC patient samples (Figure 8A). Expression of METTL3 in CRC tissues was significantly (p < 0.01) greater than that in normal tissues according to Hong Colorectal (Figure 8B) and Skrzypczak Colorectal 2 data (Figure 8C) from the Oncomine database. Consistently, the expression of LDHA was also increased in CRC tissues as compared with that in normal tissues according to Hong Colorectal (Figure 8D) and Skrzypczak Colorectal 2 data (Figure 8E). Significantly increased METTL3 (Figure 8F) and LDHA (Figure 8G) was observed in patients with increased stage of CRC patients. Using the online bioinformatics tool Kaplan‐Meier plotter 33, we found that CRC patients with increased expression of METTL3 (Figure 8H) and LDHA (Figure 8I) had significant reduced overall survival (OS) than that of their corresponding low expression patients. All these data confirmed that positive association between METTL3/LDHA axis and clinical progression of CRC patients.

## Discussion

The acquired therapeutic resistance to chemotherapy drugs such as 5-FU is the major cause for CRC treatment failure. It has been revealed that chemoresistant cells can reprogram metabolic profiles such as glycolysis and glutamine metabolism to suppress chemotherapy efficiency 34. As to 5-FU resistant CRC cells, our present study showed that the ATP generation, glucose consumption, lactate production, and OCR were increased as compared with that in parental sensitive cells. Consistently, recent evidences showed that targeting glycolysis is a novel strategy to overcome drug resistance in cancer cells 35, 36. As to CRC, exosome-delivered circRNA promotes glycolysis to induce chemoresistance of CRC cells 37. Increased expression of glycolytic enzymes was observed in sera and tissues from CRC patients displaying poor response to 5-FU-based chemotherapy 8. All these data indicated that targeting glycolysis might be a potential therapy target to overcome 5-FU resistance of CRC cells.

Our data showed METTL3-regulated m^6^A is essential for the upregulation of glycolysis and chemoresistance of CRC cells. Recent studies suggested the promotion effects of METTL3 and m^6^A in Warburg effect and aerobic glycolysis of cancer cells 22, 38, 39. For example, METTL3 stimulates the m^6^A modification of HDGF mRNA to increase its expression and then trigger the glycolysis in gastric cancer cells 40. CircPUM1 promotes cell growth and glycolysis in NSCLC via up-regulating METTL3 expression through miR-590-5p 41. As to CRC cells, METTL3 induced-CRC tumorigenesis depends on cell glycolysis in multiple CRC models 19. All these data supported the critical roles of METTL3 in the cancerous Warburg effect. Consistently, the promotion effects of METTL3 and m^6^A in chemotherapy resistance were also observed in recent investigations 42. Jin D et al reported that METTL3 activated YAP translation and induced NSCLC chemoresistance and metastasis 43, 44. Knockdown of METTL3 can increase tumor cell sensitivity to radiotherapy and chemotherapy 45, 46. Our data confirmed that inhibition of METTL3 can suppress the glycolysis and restore chemosensitivity of CRC cells.

Our data showed that LDHA, which catalyzes the conversion of pyruvate to lactate to promote glycolysis, mediates METTL3-regulated glycolysis and 5-FU resistance of CRC cells. Mechanistically, METTL3 can stabilize mRNA stability of HIF-1α to increase its expression, thus promoting the transcription of LDHA. Further, METTL3-inudced methylation of LDHA CDS region of mRNA can trigger the translation and increase the expression of LDHA. LDHA has been widely recognized as a therapeutic anticancer target 47. Previous studies indicated that LDHA was remarkedly upregulated in 5-FU resistant GC cells 48. Consistently, silencing LDHA can effectively overcome 5-FU resistance of gastric 48 and cervical 49 cancer cells. Our *in vitro* and *in vivo* data confirmed that inhibition of LDHA and METTL3 can synergistically increase *in vivo* 5-FU sensitivity of CRC resistant cells.

Collectively, our data present study indicated that METTL3 is upregulated in CRC 5-FU resistant cell and enhances the expression of LDHA to trigger glycolysis (Figure 8J). Mechanistically, METTL3 can increase the transcription of LDHA via stabilizing mRNA of HIF-1α, further, METTL3 also triggers the translation of LDHA mRNA via methylation of its CDS region and recruitment of YTHDF1. Our study provided that METTL3/LDHA axis-induced glucose metabolism should be a potential therapy target to overcome 5-FU resistance in CRC cells.

## Materials and methods

### Cell line and cell culture

Human CRC cells including HCT-116, SW480, and SW620 were purchased from the Chinese Academy of Sciences and maintained in our lab. Cells were cultured in Dulbecco's modified eagle medium (DMEM) (Invitrogen Life Technologies) with 10% fetal bovine serum (FBS, Gibco, Carlsbad, CA, USA), 1% L-glutamine and 1% penicillin/streptomycin in humidified air with 5% CO2 at 37 °C.

To induce the 5-FU resistant CRC cells, CRC cells were treated with increasing concentrations of 5-FU for about 6 months 50. Finally, the 5-FU resistant cells were named as HCT-116/5-FU, SW480/5-FU, and SW620/5-FU, respectively. The resistant cells were reselected with 5-FU every 3 months or 5-7 passage. The 5-FU resistant CRC cells were cultured with 1 μM 5-FU and replaced with no 5-FU full medium three days before experiments.

### Cell viability assay

The cell viability was evaluated by use of the Cell Counting Kit-8 (CCK-8, Dojindo Laboratories, Kumamoto, Japan) according to the instructions. Briefly, cells were treated as indicated in figure legends and then sub-cultured in a 96-well plate at 4×10^3^ cells/well. At the end of experiments, 100 µL of each culture medium containing CCK-8 reagent was added into each well. Absorbance of each well was detected at 450 nm with a microplate reader (Bio-Rad, Hercules, CA, USA). The IC_50_ values were calculated as the cell viability was inhibited to 50% by use of GraphPad Prism 6.0 (GraphPad Software Inc., San Diego, CA, USA). All data were represented based on three independent experiments.

### Glucose consumption, lactate production, and ATP Assay

Both parental and 5-FU resistant CRC cells were seeded into 6-well plates and cultured for 24 h. Then culture medium and cells were collected separately. The glucose assay kit (Applygen, Beijing, China), the lactic acid assay (BioVision, Milpitas, CA, USA), and the bioluminescent ATP assay kit (Beyotime, #S0027) were used to measure the glucose consumption, lactate production, and ATP concentration according to the manufacturers' protocols, respectively. The relative levels of glucose consumption, lactate production, and ATP concentration were divided by the number of cells. Each experiment was repeated three times in triplicates.

### Measurement of oxygen consumption rate (OCR)

The OCR was measured with the Seahorse XF bioenergetic assay according to the previous study 51 by use of the Seahorse Cell Mito Stress Test Kit (Seahorse Bioscience, North Billerica, MA, USA). Briefly, cells were seeded in the Seahorse cell plate and incubated with DMEM supplemented with 2% FBS for 12 h. The OCR was measured at a steady state and added with 1 μM of oligomycin, 1 μM of FCCP, and 1 μM of rotenone/antimycin A to obtain the maximal and non-mitochondrial respiration rates. The real-time OCR were obtained and normalized to protein concentration.

### LC-MS/MS assay for m^6^A quantification

The m^6^A quantification was conducted according to the previous studies 22, 29. Briefly, mRNA was purified from total RNA by use of biotinylated poly(dT) oligo (NEB, USA). After digested by nuclease P1 (Sigma, USA) and alkaline phosphatase (Sigma, USA), the nucleosides of m^6^A and A were separated by reverse phase ultra-performance liquid chromatography on a C18 column and analyzed by mass spectrometry. The ratio of m^6^A to A was calculated based on the standard cures.

### Real time PCR

Real time PCR was used to evaluate the expression of mRNA according to previous studies 52. The sequences of primers were:

The primers crossing exon 1 and the following intron were used to measure the levels of LDHA precursor mRNA. The sequences were: Forward: 5'ATT CCC GAT TCCC TTT TGG TT 3'; Reverse: 5'TTC ATC TGC CAA GTC CTT CA'. The expression of mRNA was normalized to the relative levels of β-actin with 2^-ΔΔCq^ method 53.

### Western blot analysis

The procedures for western blot analysis and protein visualization were performed according to our previous studies 52, 54. Primary antibodies included anti-Mettl3 (ab195352, Abcam), anti-LDHA (ABN311 - EMD Millipore), and anti-YTHDF1 (ab99080, Abcam). Anti-α-tubulin (66031-1-Ig, Proteintech) was used as a loading control. The signals were detected by enhanced chemiluminescence using a Chemidoc XRS Molecular Imager (Bio-Rad Laboratories Inc.). Quantity One software (version 4.3.0, Bio-Rad Laboratories, Inc.) was used for densitometric analysis.

### Plasmids, sh-RNA and transfection

The cDNAs of METTL3, LDHA, HIF-1α and YTHDF1 were subcloned into pcDNA3.1 to generate plasmid by use of *Bam*HI/*Eco*RI. Lipo3000 (Invitrogen, Long Island, USA) was used for plasmid transfection according to manufacturer's protocol. To generate stable METTL3 knockdown cells, cells were transfected with control and METTL3 lentivirus-shRNA, respectively, and subsequently selected with puromycin. The transfection or knockdown efficiency was evaluated by western blot analysis and/or RT-PCR.

### Dual luciferase assay

The transcriptional activity of LDHA promoter was measured by dual luciferase assay according to procedures described previously 50, 55. The region of LDHA promoter (-1000 to -1 bp) were subcloned into luciferase promoter to construct pTL-LDHA. Both pTL-LDHA and pBABE-puro were used to transfect cells. The relative promoter activity was measured by normalized values of F-Luc to that of R-Luc.

pmirGLO plasmid was used to evaluate the potential roles of LDHA mRNA translation of F-Luc. Briefly, the cDNA regions of LDHA were subcloned to pmirGLO plasmid. After transfection with pmirGLO-LDHA for 24 h, the values of F-Luc/R-Luc were measured by dual luciferase assay. The relative values of F-Luc mRNA or R-Luc mRNA was checked by qRT-PCR.

### mRNA stability assay

Cells were incubated full medium containing 5 μg/ml RNA synthesis inhibitor actinomycin D (Act-D, Sigma-Aldrich). At different time points, RNAs were extracted and then mRNA of target gene was checked by qRT-PCR.

### Protein stability assay

The protein stability was assayed by use of cycloheximide- (CHX-) chase assay. Cells were incubated with 100 μg/ml cycloheximide (CHX, #HY-12320, MedChemExpress), and then protein were extracted at different time points. The expression of LDHA was checked by western blot analysis.

### m^6^A-RIP-PCR

The m^6^A-RIP-PCR was conducted by use of Magna MeRIP™ m^6^A Kit (Millipore, MA) according to the manufacturer's protocol. Total RNA was randomly fragmented with chemical reagents treatment, m^6^A RNAs were immunoprecipitated with m^6^A antibody (Synaptic Systems) and Dynabeads® Protein A (ThermoFisher Scientific). The RNA-antibody-conjugated beads was eluted with 100 µl Elution Buffer (75nM NaCl, 50 nM Tris-HCl, pH 7.5, 6.25 nM EDTA, 1% (w/v) SDS, 20 mg/ml Proteinase K). The eluted RNA was recovered by ethanol precipitation, reverse transcribed and quantification by qPCR. House-keeping gene HPRT1 was chosen as internal control since HPRT1 mRNA did not have m^6^A peaks from m^6^A profiling data 29.

### RIP-PCR

The RIP-PCR was conducted according to the previous study 22. Cells were irradiated twice with 400 mJ/cm^2^ at 254 nm by Stratalinker on ice and lysed. The supernatant was pre-cleared with Dynabeads protein A/G beads and further incubated with different antibodies (anti- anti-YTHDF1 (ab99080, Abcam), Anti-IGF2BP1 (8482S, Cell Signaling); Anti-IGF2BP2 (14672S, Cell Signaling); Anti-IGF2BP3 (25864S, Cell Signaling)) or IgG-conjugated Protein A/G Magnetic Beads with RNase inhibitors at 4 °C overnight. The bound RNAs were immunoprecipitated with beads and extracted with TRIzol. IP enrichment ratio of a transcript was calculated as ratio of its amount in IP to that in the input, yielded from same amounts of cells.

### Experimental animals and xenograft models

All animal experiments complied with Institutional Animal Care and Use Committee in our university. BALB/c nude mice (5 weeks old) were purchased from the Beijing HFK Bioscience Co. Ltd. For subcutaneous transplanted model, sh-control and* sh-METTL3* HCT-116/5-FU cells (5 × 10^6^ per mouse) were diluted in 100μL PBS + 100 μL Matrigel (BD Biosciences, San Jose, CA, USA) and injected subcutaneously in the rear flank fat pad of the nude mice. When the tumor was visible, mice were randomized into four groups of five mice with similar average xenograft tumor volumes and assigned to a receive treatment with 5-FU (50 mg/kg in 10% (2-hydroxypropyl)-β-cyclodextrin, b.i.d.) combined with or without FX11 (50 mg/kg) every day for 10 days. Tumor growth was measured twice weekly using calipers, with the tumor volume (mm^3^) calculated using the following formula: V = L (length) × W (width)^2^/2.

### Database (DB) analysis

The expression of LDHA and METTL3 in CRC cancers were analyzed by using data obtained from the Oncomine Database (www.oncomine.org). The expression profiles of LDHA and METTL3 among the subtypes of CRC patients were downloaded from LinkedOmics (http://www.linkedomics.org), which is a publicly available portal that includes multi-omics data from all of 32 cancer types from The Cancer Genome Atlas (TCGA) project. The protein levels of METTL3 and LDHA were downloaded from the project of “CPTAC Colon Cancer Confirmatory Study” (BioProject Accession: PRJNA514017 ID: 514017, https://cptac-data-portal.georgetown.edu/study-summary/S045). We used Kaplan-Meier database (http://kmplot.com/analysis/) to test Overall survival (OS) of LDHA and METTL3.

### Statistical analysis

Experiment was repeated three times or specified at figure legends. Data were presented as mean ± standard deviation (Sd). The student's t-test (two-tailed) or the analysis of one-way ANOVAs were used to check the differences. P ≤ 0.05 were recognized as significant.

## Figures and Tables

**Table 1:** Primer sequences

Gene	RT-Primer
METTL3	CTATCTCCTGGCACTCGCAAGA
	GCTTGAACCGTGCAACCACATC
FTO	CCAGAACCTGAGGAGAGAATGG
	CGATGTCTGTGAGGTCAAACGG
ALKBH5	CGCTGCCGCCGAACCTTA
	GGATGCCGCTCTTCACCTTGC
GLUT1	F: GGCCAAGAGTGTGCTAAAGAA
	R: ACAGCGTTGATGCCAGACAG
GLUT2	F: GCTGCTCAACTAATCACCATGC
	R: TGGTCCCAATTTTGAAAACCCC
HK2	F: GAGCCACCACTCACCCTACT
	R: CCAGGCATTCGGCAATGTG
GPI	F: GGAGACCATCACGAATGCAGA
	R: TAGACAGGGCAACAAAGTGCT
PFK1	F: AGCGTTTCGATGATGCTTCAG
	R: GGAGTCGTCCTTCTCGTTCC
ALDOA	F: CAGGGACAAATGGCGAGACTA
	R: GGGGTGTGTTCCCCAATCTT
GAPDH	F: GGAGCGAGATCCCTCCAAAAT
	R: GGCTGTTGTCATACTTCTCATGG
PGK1	F: TGGACGTTAAAGGGAAGCGG
	R: GCTCATAAGGACTACCGACTTGG
PGAM1	F: GTGCAGAAGAGAGCGATCCG
	R: CGGTTAGACCCCCATAGTGC
ENO1	F: TGGTGTCTATCGAAGATCCCTT
	R: CCTTGGCGATCCTCTTTGG
PK	F: ATAACGCCTACATGGAAAAGTGT
	R: TAAGCCCATCATCCACGTAGA
LDHA	F: ATGGCAACTCTAAAGGATCAGC
	R: CCAACCCCAACAACTGTAATCT
LDHB	F: TGGTATGGCGTGTGCTATCAG
	R: TTGGCGGTCACAGAATAATCTTT
FOXM1	CGTCGGCCACTGATTCTCAAA
	GGCAGGGGATCTCTTAGGTTC
HIF-1α	GAACGTCGAAAAGAAAAGTCTCG
	CCTTATCAAGATGCGAACTCACA
HIF-2α	CGGAGGTGTTCTATGAGCTGG
	AGCTTGTGTGTTCGCAGGAA
JMJD2A	ATCCCAGTGCTAGGATAATGACC
	ACTCTTTTGGAGGAACAACCTTG
PGC1β	GATGCCAGCGACTTTGACTC
	ACCCACGTCATCTTCAGGGA
18s rRNA	CGGACAGGATTGACAGATTGATAGC
	GCGTCCTCCTGGCTGAAGTGG
β-actin	CATGTACGTTGCTATCCAGGC
	CTCCTTAATGTCACGCACGAT

## References

[1] Siegel RL, Miller KD, Jemal A (2019). Cancer statistics, 2019. *CA Cancer J Clin*.

[2] Vodenkova S, Buchler T, Cervena K, Veskrnova V, Vodicka P, Vymetalkova V (2020). 5-fluorouracil and other fluoropyrimidines in colorectal cancer: Past, present and future. *Pharmacol Ther*.

[3] Vertessy BG, Toth J (2009). Keeping uracil out of DNA: physiological role, structure and catalytic mechanism of dUTPases. *Acc Chem Res*.

[4] Hammond WA, Swaika A, Mody K (2016). Pharmacologic resistance in colorectal cancer: a review. *Ther Adv Med Oncol*.

[5] Leggett B, Whitehall V (2010). Role of the serrated pathway in colorectal cancer pathogenesis. *Gastroenterology*.

[6] Zahan T, Das PK, Akter SF, Habib R, Rahman MH, Karim MR (2020). Therapy Resistance in Cancers: Phenotypic, Metabolic, Epigenetic and Tumour Microenvironmental Perspectives. *Anticancer Agents Med Chem*.

[7] Bhattacharya B, Low SHH, Soh C, Mustapa NK, Beloueche-Babari M, Koh KX (2014). Increased drug resistance is associated with reduced glucose levels and an enhanced glycolysis phenotype. *Brit J Pharmacol*.

[8] Shin YK, Yoo BC, Hong YS, Chang HJ, Jung KH, Jeong SY (2009). Upregulation of glycolytic enzymes in proteins secreted from human colon cancer cells with 5-fluorouracil resistance. *Electrophoresis*.

[9] Jiang JX, Gao S, Pan YZ, Yu C, Sun CY (2014). Overexpression of microRNA-125b sensitizes human hepatocellular carcinoma cells to 5-fluorouracil through inhibition of glycolysis by targeting hexokinase II. *Mol Med Rep*.

[10] Xuan Y, Hur H, Ham IH, Yun J, Lee JY, Shim W (2014). Dichloroacetate attenuates hypoxia-induced resistance to 5-fluorouracil in gastric cancer through the regulation of glucose metabolism. *Exp Cell Res*.

[11] Park JH, Pyun WY, Park HW (2020). Cancer Metabolism: Phenotype, Signaling and Therapeutic Targets. *Cells*.

[12] Urbanska K, Orzechowski A (2019). Unappreciated Role of LDHA and LDHB to Control Apoptosis and Autophagy in Tumor Cells. *Int J Mol Sci*.

[13] Komurov K, Tseng JT, Muller M, Seviour EG, Moss TJ, Yang L (2012). The glucose-deprivation network counteracts lapatinib-induced toxicity in resistant ErbB2-positive breast cancer cells. *Mol Syst Biol*.

[14] Li G, Li Y, Wang DY (2021). Overexpression of miR-329-3p sensitizes osteosarcoma cells to cisplatin through suppression of glucose metabolism by targeting LDHA. *Cell Biol Int*.

[15] Hu J, Huang L, Ding Q, Lv J, Chen Z (2021). Long noncoding RNA HAGLR sponges miR-338-3p to promote 5-Fu resistance in gastric cancer through targeting the LDHA-glycolysis pathway. *Cell Biol Int*.

[16] Ponnusamy L, Mahalingaiah PKS, Singh KP (2020). Epigenetic reprogramming and potential application of epigenetic-modifying drugs in acquired chemotherapeutic resistance. *Adv Clin Chem*.

[17] Roundtree IA, Evans ME, Pan T, He C (2017). Dynamic RNA Modifications in Gene Expression Regulation. *Cell*.

[18] Yu H, Zhao K, Zeng HJ, Li ZW, Chen K, Zhang ZX (2021). N-6-methyladenosine (m(6)A) methyltransferase WTAP accelerates the Warburg effect of gastric cancer through regulating HK2 stability. *Biomed Pharmacother*.

[19] Shen CQ, Xuan BQ, Yan TT, Ma YR, Xu PP, Tian XL (2020). m(6)A-dependent glycolysis enhances colorectal cancer progression. *Mol Cancer*.

[20] Qing Y, Dong L, Gao L, Li C, Li Y, Han L (2021). R-2-hydroxyglutarate attenuates aerobic glycolysis in leukemia by targeting the FTO/m(6)A/PFKP/LDHB axis. *Mol Cell*.

[21] Wang Y, Lu JH, Wu QN, Jin Y, Wang DS, Chen YX (2019). LncRNA LINRIS stabilizes IGF2BP2 and promotes the aerobic glycolysis in colorectal cancer. *Mol Cancer*.

[22] Li ZH, Peng YX, Li JX, Chen ZJ, Chen F, Tu J (2020). N-6-methyladenosine regulates glycolysis of cancer cells through PDK4. *Nat Commun*.

[23] Yu H, Yang X, Tang JY, Si SH, Zhou ZJ, Lu JC (2021). ALKBH5 Inhibited Cell Proliferation and Sensitized Bladder Cancer Cells to Cisplatin by m6A-CK2 alpha-Mediated Glycolysis. *Mol Ther-Nucl Acids*.

[24] Lukey MJ, Katt WP, Cerione RA (2018). Targeting Therapy Resistance: When Glutamine Catabolism Becomes Essential. *Cancer Cell*.

[25] Wicki A, Mandala M, Massi D, Taverna D, Tang HF, Hemmings BA (2016). Acquired Resistance to Clinical Cancer Therapy: A Twist in Physiological Signaling. *Physiological Reviews*.

[26] Alptekin A, Ye B, Ding HF (2017). Transcriptional Regulation of Stem Cell and Cancer Stem Cell Metabolism. *Curr Stem Cell Rep*.

[27] Chen F, Chen Z, Guan T, Zhou Y, Ge L, Zhang H (2021). N(6) -Methyladenosine Regulates mRNA Stability and Translation Efficiency of KRT7 to Promote Breast Cancer Lung Metastasis. *Cancer Res*.

[28] Wang X, Zhao BS, Roundtree IA, Lu Z, Han D, Ma H (2015). N(6)-methyladenosine Modulates Messenger RNA Translation Efficiency. *Cell*.

[29] Wang X, Lu Z, Gomez A, Hon GC, Yue Y, Han D (2014). N6-methyladenosine-dependent regulation of messenger RNA stability. *Nature*.

[30] Firth JD, Ebert BL, Ratcliffe PJ (1995). Hypoxic regulation of lactate dehydrogenase A. Interaction between hypoxia-inducible factor 1 and cAMP response elements. *J Biol Chem*.

[31] Jiang L, Li Y, He Y, Wei D, Yan L, Wen H (2021). Knockdown of m6A Reader IGF2BP3 Inhibited Hypoxia-Induced Cell Migration and Angiogenesis by Regulating Hypoxia Inducible Factor-1alpha in Stomach Cancer. *Front Oncol*.

[32] Zaccara S, Ries RJ, Jaffrey SR (2019). Reading, writing and erasing mRNA methylation. *Nat Rev Mol Cell Biol*.

[33] Wu H, Wang Y, Ying M, Jin C, Li J, Hu X (2021). Lactate dehydrogenases amplify reactive oxygen species in cancer cells in response to oxidative stimuli. *Signal Transduct Target Ther*.

[34] Belisario DC, Kopecka J, Pasino M, Akman M, De Smaele E, Donadelli M (2020). Hypoxia Dictates Metabolic Rewiring of Tumors: Implications for Chemoresistance. *Cells*.

[35] Cheng C, Xie Z, Li Y, Wang J, Qin C, Zhang Y (2018). PTBP1 knockdown overcomes the resistance to vincristine and oxaliplatin in drug-resistant colon cancer cells through regulation of glycolysis. *Biomed Pharmacother*.

[36] Xu RH, Pelicano H, Zhou Y, Carew JS, Feng L, Bhalla KN (2005). Inhibition of glycolysis in cancer cells: a novel strategy to overcome drug resistance associated with mitochondrial respiratory defect and hypoxia. *Cancer Res*.

[37] Wang X, Zhang H, Yang H, Bai M, Ning T, Deng T (2020). Exosome-delivered circRNA promotes glycolysis to induce chemoresistance through the miR-122-PKM2 axis in colorectal cancer. *Mol Oncol*.

[38] Wang QQ, Guo XC, Li L, Gao ZH, Su XK, Ji M (2020). N-6-methyladenosine METTL3 promotes cervical cancer tumorigenesis and Warburg effect through YTHDF1/HK2 modification. *Cell Death Dis*.

[39] Lin Y, Wei X, Jian Z, Zhang X (2020). METTL3 expression is associated with glycolysis metabolism and sensitivity to glycolytic stress in hepatocellular carcinoma. *Cancer Med*.

[40] Wang Q, Chen C, Ding Q, Zhao Y, Wang Z, Chen J (2019). METTL3-mediated m(6)A modification of HDGF mRNA promotes gastric cancer progression and has prognostic significance. *Gut*.

[41] Li M, Wang Q, Zhang X, Yan N, Li X (2021). CircPUM1 promotes cell growth and glycolysis in NSCLC via up-regulating METTL3 expression through miR-590-5p. *Cell Cycle*.

[42] Qiao X, Zhu L, Song R, Shang C, Guo Y (2021). METTL3/14 and IL-17 signaling contribute to CEBPA-DT enhanced oral cancer cisplatin resistance. *Oral Dis*.

[43] Jin D, Guo J, Wu Y, Du J, Yang L, Wang X (2019). m(6)A mRNA methylation initiated by METTL3 directly promotes YAP translation and increases YAP activity by regulating the MALAT1-miR-1914-3p-YAP axis to induce NSCLC drug resistance and metastasis. *J Hematol Oncol*.

[44] Xu Z, Peng B, Cai Y, Wu G, Huang J, Gao M (2020). N6-methyladenosine RNA modification in cancer therapeutic resistance: Current status and perspectives. *Biochem Pharmacol*.

[45] Taketo K, Konno M, Asai A, Koseki J, Toratani M, Satoh T (2018). The epitranscriptome m6A writer METTL3 promotes chemo- and radioresistance in pancreatic cancer cells. *Int J Oncol*.

[46] Uddin MB, Roy KR, Hosain SB, Khiste SK, Hill RA, Jois SD (2019). An N(6)-methyladenosine at the transited codon 273 of p53 pre-mRNA promotes the expression of R273H mutant protein and drug resistance of cancer cells. *Biochem Pharmacol*.

[47] Feng Y, Xiong Y, Qiao T, Li X, Jia L, Han Y (2018). Lactate dehydrogenase A: A key player in carcinogenesis and potential target in cancer therapy. *Cancer Med*.

[48] Hu J, Huang LJ, Ding Q, Lv JM, Chen Z (2021). Long noncoding RNA HAGLR sponges miR-338-3p to promote 5-Fu resistance in gastric cancer through targeting the LDHA-glycolysis pathway. *Cell Biol Int*.

[49] Shao XC, Zheng XH, Ma D, Liu Y, Liu GY (2021). Inhibition of lncRNA-NEAT1 sensitizes 5-Fu resistant cervical cancer cells through de-repressing the microRNA-34a/LDHA axis. *Bioscience Rep*.

[50] Chen Y, Zhang K, Li Y, Guo R, Zhang K, Zhong G (2019). Oestrogen-related receptor alpha mediates chemotherapy resistance of osteosarcoma cells via regulation of ABCB1. *J Cell Mol Med*.

[51] Zhou C, Lyu LH, Miao HK, Bahr T, Zhang QY, Liang T (2020). Redox regulation by SOD2 modulates colorectal cancer tumorigenesis through AMPK-mediated energy metabolism. *Mol Carcinogen*.

[52] Yin L, Yang Y, Zhu W, Xian Y, Han Z, Huang H (2021). Heat Shock Protein 90 Triggers Multi-Drug Resistance of Ovarian Cancer via AKT/GSK3beta/beta-Catenin Signaling. *Front Oncol*.

[53] Chen Y, Zhao H, Li H, Feng X, Tang H, Zhang J (2019). LINC01234/MicroRNA-31-5p/MAGEA3 Axis Mediates the Proliferation and Chemoresistance of Hepatocellular Carcinoma Cells. *Mol Ther Nucleic Acids*.

[54] Yang Y, Jiang H, Li W, Chen L, Zhu W, Xian Y (2020). FOXM1/DVL2/Snail axis drives metastasis and chemoresistance of colorectal cancer. *Aging (Albany NY)*.

[55] Chen Y, Zhang K, Li Y, He Q (2017). Estrogen-related receptor alpha participates transforming growth factor-beta (TGF-beta) induced epithelial-mesenchymal transition of osteosarcoma cells. *Cell Adh Migr*.

